# Immune-Protective Formulations and Process Strategies for Improved Survival and Function of Transplanted Islets

**DOI:** 10.3389/fimmu.2022.923241

**Published:** 2022-07-12

**Authors:** Yannan Shi, Ying-Zheng Zhao, Zhikai Jiang, Zeqing Wang, Qian Wang, Longfa Kou, Qing Yao

**Affiliations:** ^1^ School of Pharmaceutical Sciences, Wenzhou Medical University, Wenzhou, China; ^2^ The Second Affiliated Hospital of Wenzhou Medical University, Wenzhou, China

**Keywords:** islet transplantation, immune reactions, biomaterials, islet encapsulation, cell therapy

## Abstract

Type 1 diabetes (T1D) is an autoimmune disease caused by the immune system attacking and destroying insulin-producing β cells in the pancreas. Islet transplantation is becoming one of the most promising therapies for T1D patients. However, its clinical use is limited by substantial cell loss after islet infusion, closely related to immune reactions, including instant blood-mediated inflammatory responses, oxidative stress, and direct autoimmune attack. Especially the grafted islets are not only exposed to allogeneic immune rejection after transplantation but are also subjected to an autoimmune process that caused the original disease. Due to the development and convergence of expertise in biomaterials, nanotechnology, and immunology, protective strategies are being investigated to address this issue, including exploring novel immune protective agents, encapsulating islets with biomaterials, and searching for alternative implantation sites, or co-transplantation with functional cells. These methods have significantly increased the survival rate and function of the transplanted islets. However, most studies are still limited to animal experiments and need further studies. In this review, we introduced the immunological challenges for islet graft and summarized the recent developments in immune-protective strategies to improve the outcomes of islet transplantation.

## Introduction

Type 1 diabetes (T1D) is a chronic, immune-mediated disease. The insulin-producing β cells in the pancreas are destroyed by the autoimmune system, leading to hyperglycemia. It has been acknowledged that patients with T1D suffer from more considerable medical costs and increasing mortality and are at a high risk of developing other complications, such as chronic kidney disease, infections, osteoporosis, and cardiovascular disease (1). There is an urgent need to pursue a better therapeutic schedule and relieve the suffering of individuals with T1D patients. The main goal of T1D treatment is to maintain blood glucose at a normal range to reduce severe diabetes-associated complications. In the early 1920s, the discovery of insulin revolutionized diabetes treatment and converted a rapidly fatal disease (especially for those with T1D) to a chronic condition. Insulin therapy could help keep patients’ blood glucose within a narrow range, decreasing the risk for diabetic complications (2) and improving their overall quality of life. However, insulin-based therapy is not a perfect treatment regimen. The expensive medical cost and life-long subcutaneous insulin injections are still troubling for these patients. In addition, blood glucose monitoring, insulin dosing, diet, and exercise require strict attention. More importantly, good glycemic control is not available for all patients, and some of them even experience serious side effects of insulin therapy, including hypoglycemia and allergies.

Pancreatic β-cell replacement therapy aims to maintain normal blood glucose levels by restoring endogenous and regulated secretion of insulin and other hormones. The successful simultaneous kidney–pancreas (SPK) transplants performed in two patients with end-stage diabetic nephropathy in 1966 are effective proof of concept (3). It should be noted that pancreas transplantation is a major surgery that carries a significant risk of surgical complications and immunological rejection, most of which are related to the exocrine tissue. In contrast, islet transplantation is a less invasive alternative to transfer healthy and functional pancreatic β cells. The modern era of islet transplantation began in 1972 with the report from two laboratories demonstrating the successful reversal of diabetes in rodents (4). However, isolated islets are still susceptible to immunological rejection to some extent despite maintenance immunosuppression. After more than three decades of investigation, in 2000, the Edmonton group reported their remarkable work that 100% of patients (n=7) with labile diabetes who received islet transplantation and corticosteroid-free immunosuppression become insulin independent (5).

Indeed, the apparent effectiveness of the Edmonton protocol renewed global interest in islet transplantation as a viable T1D therapeutic option. Over time, islet transplantation has improved significantly, with numerous additional enhancements involving optimum isolation procedure, culture, securer transplant procedures, and much efficient anti-inflammatory and immunomodulatory approaches.

Although the Edmonton protocol made gradual progress, it has not entirely gotten rid of external insulin. Over time, only 10% of patients were found to be independent of external insulin over 5 years. In addition, these patients, with the treatment of islet transplantation, need systematic immunosuppressive regimens, which are associated with several side effects such as insulin resistance, nephrotoxicity, and increased risk of cancer and infections (6). Therefore, new strategies are urgently needed to avoid the lifelong use of immunosuppressive agents, improving the graft survival and secretory function.

## Challenges Associated With Islet Transplantation

**Figure 1:**
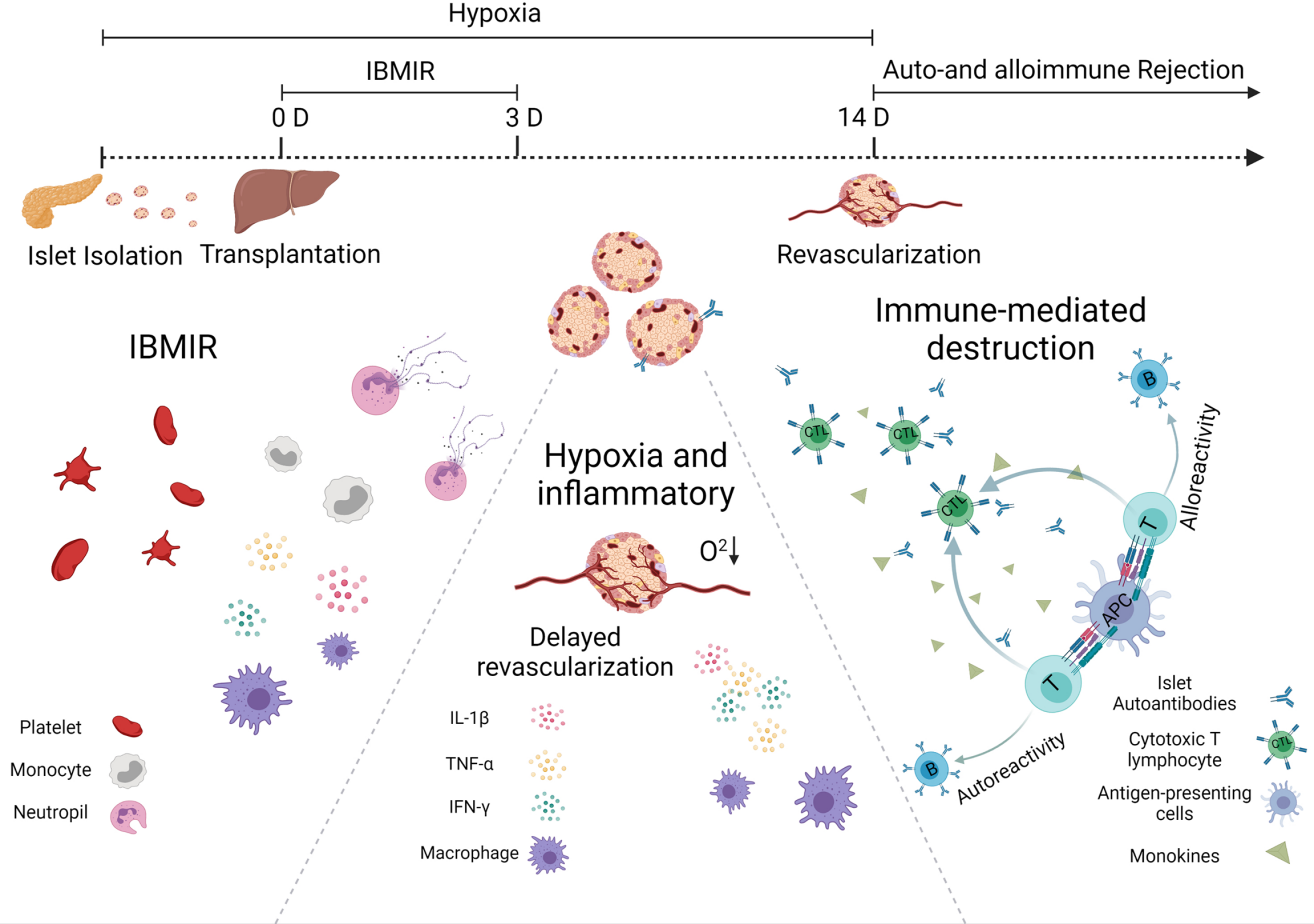
Immunological challenges associated with islet transplantation including IBMIR, hypoxic/oxidative stress, and rejection from host immune system.

From the moment they are transplanted into the body, islets would be detected by the host’s immune system, which will respond against them. As presented in **Figure 1**, islet-graft-confronted immune responses can be divided mainly into three types, including (1) autoimmune recurrence and alloimmunity, (2) instant blood-mediated inflammatory reaction (IBMIR), and (3) hypoxia and oxidative stress.

### Alloimmunity and Autoimmune Recurrence

One of the most effective processes performed by the immune system is the response against the foreign invader, including transplanted allograft. The acute and accurate rejection response is mainly due to the dendritic cells (DCs), one type of the most powerful professional antigen-presenting cells (APCs). They can express major histocompatibility complex (MHC) class I and II antigens, that is why the DCs enable to activate both CD4+ helper and CD8+ cytotoxic T cells. In general, there are three ways to prime the T cells. In a direct way, DCs can migrate from the graft islets to the secondary lymphoid organ, present donor MHC molecules, and activate the alloreactive T cells. In a semidirect pathway, DCs and other APCs can phagocytize allogeneic cells, present the donor allogenic MHC molecules on their surface, and then activate the T cell. In an indirect way, allogenic proteins are degraded by recipient APCs, and autologous MHCs present the allogenic peptides derived from them. These allopeptide–self-MHC complexes are recognized by the T-cell receptor (7). The difference between the semidirect and indirect allorecognition is whether peptides derived from allogeneic transplantation antigens are displayed on autologous MHC class II molecules expressed on autologous APCs.

The alloreactive T cells play an essential role in allograft immunity. Migration of the activated T cells to the grafts will destroy the islets. Usually, CD8+ T cells secrete cytotoxic molecules such as perforin and granzyme B to damage the islets directly, while CD4+ T cells do not generally exhibit cytotoxic activity, but it will help boost CD8+ T cells and secrete some pro-inflammatory cytokines such as tumor necrosis factor alpha (TNF-α) and interferon gamma (IFN-γ) (7). These cytokines can recruit more immune cells to reject and kill islets. They also promote macrophages to polarize into the M1 phenotype and stimulate a positive feedback loop, increasing cytokine production and killing more islets (8). Since T cells are essential for allograft rejection, current clinical immunosuppressive strategies primarily target T cells. More and more evidence suggest that B cells also play a major role in long-lasting chronic rejection of allogeneic transplantation (9). In non-human primates (NHPs), the addition of Rituximab (a monoclonal antibody that targets B cells) to anti-thymocyte globulin induction and a limited course of rapamycin successfully prolonged islets allograft survival for many years after rapamycin discontinuation (10).

T1D is a disease characterized by the destruction of insulin-secreting β cells attacked by autoimmunity. The pathogenesis might also damage newly implanted islet grafts; unlike the alloimmunity, which requires alloantigen-presenting APCs to prime T cells activation, autoimmune memory can directly reawake silent original autoreactive T cells after islet transplantation. This theory was confirmed by the fact that syngeneic islets were still largely damaged in autoimmune diabetic recipients observed in a twin-to-twin pancreas transplantation experiment (11). From another point, the precise role of autoantibodies against β-cell autoantigens, e.g., insulin-specific autoantibodies, insulinoma antigen, zinc transporter-8, and glutamic acid decarboxylase, in the pathogenesis of T1D remains unclear. However, they are the most reliable markers for assessing the autoimmune process leading to T1D. Individuals with two or more autoantibodies are more likely to develop T1D than those with only one autoantibody. Regardless of the source of β cells transplanted into a patient with T1D, autoimmune T cells would target and attack the newly implanted insulin-secreting cells. Strategies that inhibit or remove autoimmune T cells have been adopted to prevent this from happening to facilitate long-term transplanted islet function (12). Therefore, autoreactive T cells (especially CD4 and CD8 T cells) play a central role in the apoptotic β-cell destruction and could act as viable therapy intervention targets.

Both alloimmune and autoimmune responses contributed to the decrease in the graft’s survival, but which one represents a more significant obstacle to the success of the islet transplantation currently remains unknown.

### Instant Blood-Mediated Inflammatory Reaction

So far, intraportal islet transplantation infusion remains the leading choice for patients that need islet replacement in clinic. However, the profound islet attrition that occurs in the immediate post-transplant period blocks the success of this regimen. One primary reason for the loss of islets is termed instant blood-mediated inflammatory reaction (IBMIR). The pathomechanism of IBMIR is the thrombotic/inflammatory cascade that starts with the activation of coagulation and complement system. An *in vitro* vascular model indicated that the islets appeared to clot in 5 mins after contacting the blood (13). Rapid platelets binding onto the islet surface often cause a significant reduction of platelet in the blood and promote fibrin formation around the islet graft. In addition, those bound platelets presented upregulated expression of p-selectin and β-thromboglobulin, indicating that these platelets have been activated and this platelet-activated response could directly happen on the transplanted islets. During the first 5 min, a rapid insulin release was observed as platelets bonded to the surface of the islets. Initially, scientists believed that the complement-mediated damage mainly causes insulin secretion. However, the short occurrence time and the lack of complement activation byproducts object to this hypothesis. The most likely reason is that the activated platelets release factors such as Ca^2+^, ATP, and ADP, which are stimulated following insulin release (14). In addition, during this process, released inflammatory cytokines also contributed to the apoptosis and necrosis of transplanted islets. In addition to causing direct islet loss, IBMIR can also promote antigen presentation, leading to an accelerated and enhanced cell-mediated immune response in the later stage of islet transplantation (15).

Different approaches have been followed to protect islet grafts from detrimental IBMIR, including inhibiting coagulation, complement activation, and leukocyte recruitment (16). Specifically, these approaches could be achieved by using soluble inhibitors, islet surface modification, and adding assistant functional cells (17, 18). The glycosaminoglycan low-molecular weight dextran sulfate (LMW-DS) can inhibit both complement and coagulation cascades (19). The LMW-DS inhibited macroscopic clotting and IBMIR in cynomolgus monkey models (20). CD39 is a critical thromboregulatory molecule expressed on the luminal surface of quiescent endothelial cells, which could limit platelet activation. Karen et al. found that when incubated with human blood, islets isolated from CD39 transgenic mice significantly delayed clotting time compared to wild-type islets (21). In addition, the surface modification of islets to avoid direct contact with blood or immune cells might be another feasible strategy. This technique could prolong the graft survival compared with bare islets in the liver of diabetic mice without doing harm to the secretory function (22).

### Hypoxia, Oxidative Stress, and Inflammatory Reactions

ROS is the byproduct of oxidative phosphorylation, produced from various sources in cells, including xanthine oxidase, cytochrome 450, and mitochondria. The electron transport chain (ETC), consisting of five multi-subunit protein complexes (complexes I–V), is located in the inner mitochondrial membrane. During phosphorylation, approximately 1%–2% of O_2_ reacts with electrons leaked from complex I and III, leading to partial reduction of O_2_ and superoxide generation (O_2_^−^). Due to the high rate of O_2_ consumption in mitochondria, it is believed that mitochondria are the primary source of ROS in cells. Mitochondria is not only the critical organelles for cellular metabolism but also the important O_2_ sensing detector. In response to hypoxia, excessive ROS are released from complex III, which has the ability to stabilize hypoxia-inducible factor 1 alpha (HIF-1α). Hypoxia could also stimulate ROS production from complex I, which also contributes to HIF-1 stabilization (23). In normoxia conditions, HIF-1α is oxidized (hydroxylated) by prolyl hydroxylases (PHDs) with α-ketoglutarate derived from the tricarboxylic acid (TCA) cycle, which becomes ubiquitinated, then catabolized by proteasomes. Such HIF-1α is continuously synthesized and degraded. Under hypoxia, the stabilized HIF-1α is translocated into the nucleus and dimerizes with HIF-1β and, in turn, binds to a core hypoxia response element in a wide array of genes involved in a diversity of biological processes and directly transactivates glycolytic enzyme genes. This HIF-mediated adaptation to hypoxia is important for cell survival. However, ROS are highly reactive. Excessive ROS can cause oxidative damage to lipids, proteins, and nucleic and elicits cell apoptotic cell death (24).

In the early stage of transplantation, the survival of transplanted islets is highly dependent upon oxygen supply. According to reports in the literature, islets that account for only 1% of the total pancreatic mass receive about 10%–15% of arterial blood (15), which indicates that the amount of O_2_ per islet volume that is transported by hemoglobin is much larger than that of another pancreatic parenchyma. This phenomenon also suggests that islets are more dependent on high oxygen than normal cells. Thus, it is not hard to understand that islets are more vulnerable toward hypoxia than other cells. In healthy tissues in the physiological state, ROS could be neutralized by effective intracellular antioxidant systems. However, islets usually possess a weak antioxidant defense system and hold poor capacity to scavenge ROS and other free radicals, making them particularly susceptible to hypoxia and following oxidative stress (25). During the isolation procedure, enzymatic and mechanical digestion could both harm the delicate pancreatic islets. After transplantation, the process of revascularization usually requires more than 10 days, while complete vascular remodeling can take up to 3 months (26). In the meantime, islet survival mainly depends on the passive diffusion of nutrients and oxygen, which is far from enough. The islets are subjected to hypoxia throughout the process from isolation to transplantation and might cause substantial islet loss.

The extreme hypoxia environment leads to the mass necrosis of islets, triggering the innate immune response. Although the mechanism has not been fully explored, recent studies suggest Toll-like receptors (TLRs)-related pathways might play an essential role in this process (27). TRLs are a family of pattern recognition receptors that bind to endogenous ligands released by damaged cells (damage-associated molecular patterns, DAMPs), which can be released in large quantities by the mass apoptotic islets early after transplantation (28). The TLR signaling pathway will ultimately lead to the production of inflammatory cytokines through the activation of the nuclear factor-κB (NF-κB) transcription factor. In addition, the TLRs also participate in the pathogenesis of allogeneic transplant rejection. All TLRs, except TLR3, initiate myeloid differentiation primary response gene88 (MyD88)-dependent signaling, often occurring in antigen-presenting cells (APCs). In APCs, the MyD88-dependent pathway regulates cell maturation, characterized by the increased expression of CD80, CD86, MHC class II, and inflammatory cytokines, e.g., TNFα, interleukin (IL)-6, and IL-12. These events collectively contributed to enhanced T-cell stimulation and allograft rejection (29).

## Strategies on How Transplanted Islets Achieve Immune Protection

**Figure 2:**
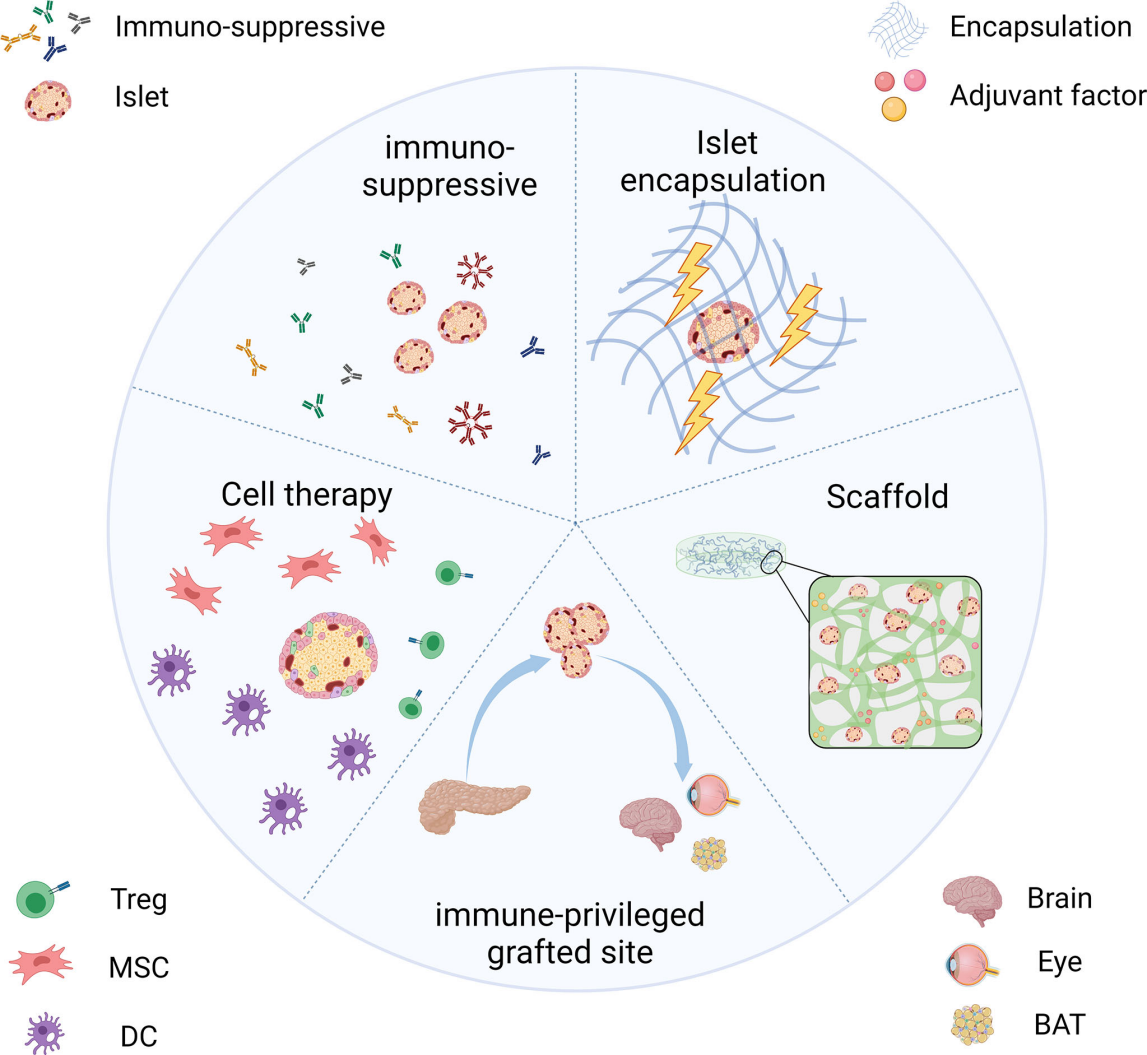
Strategies for transplanted islets achieving immune protection include the use of immunosuppressive inducing therapeutics, islet encapsulation/scaffold to provide an immune isolate environment, choosing the immune-privileged grafted site, and co-transplantation with immunomodulatory cells.

The above section describes the responses of the host immune system toward the transplanted islets. Many pieces of research have been carried out in human or non-human models to solve the problems. We outlined recent strategies for keeping the immune functioning while the grafts can evade the host immune system (**Figure 2**). Among these approaches, the use of immunosuppressive agents is trying to solve the rejection in islet transplantation by inducing immune tolerance. Encapsulation and scaffold are based on pharmaceutical science that isolates islets from various immune cells and provides them with an appropriate microenvironment. Alternatively, we could also choose an alternative immunological privileged site, anatomically isolated from immune cells or has mechanisms to suppress the immune response in their local microenvironment.

### Use of Immunosuppressive-Inducing Therapeutics

Solid-organ and cellular transplantation require lifelong immunosuppressive regimes, increasing the risk of infection and malignancy. For all kinds of transplantation procedures, the balance between efficacy and toxicity must be sought. Immunosuppression includes an induction phase during the transfusion period and a maintenance phase during the entire period of islet transplantation. A further difficulty with islet transplantation is that most current immune-suppressive agents damage β cells or induce peripheral insulin resistance (30). It is hard to find an optional immunosuppressive strategy to overcome alloimmune and recurrent autoimmune reactions without harming the grafts.

The immunosuppressive strategies in the early development of clinical islet transplantation were proposed based on solid organ transplantation because the islet transplants were usually combined with kidney allografts in that period. Hence, most islet transplanted cases before the Edmonton protocol choose the immunosuppression regimen consisting of a combination of corticosteroids, azathioprine, and cyclosporine (31). They seem to be the cornerstone of the immunosuppressive agents for transplanted rejection. However, due to their nephrotoxicity, inhibition of islet function, and potential for diabetogenic consequences, they are far from ideal candidates for islet transplantation.

The Edmonton protocol in 2000 was a milestone in the process of islet transplantation. The team transplanted islets from human donors to the liver of seven patients *via* the portal vein. To avoid immune rejection to the allogenic islets, they design an effective immunosuppressive regimen including sirolimus, low dosage of tacrolimus, and daclizumab against IL-2 receptors instead of traditional glucocorticoid (5). All recipients maintained normoglycemia for 3 years without extra insulin treatment; some achieved glucose homeostasis over 5 years.

The current available immunosuppressive agents can be broadly divided into two categories, the maintenance and the induction of immunosuppression. The principle of the maintenance immunosuppression is regarding lifelong inhibition of immune cells activation and proliferation, such as the calcineurin inhibitor (tacrolimus) used in the Edmonton protocol, with the clear disadvantage that most of these agents show liver/kidney toxicity and have direct toxicity to β cells. The future of achieving a permanent state of tolerance to the islet grafts without chronic immunosuppressive treatment is the pursuit of induced immunosuppression. The induced immunosuppression is adopting preemptive methods to boost the consumption of immune cells or inhibit the cell activation prior to islet transplantation. Since inhibiting T cells can decrease both humoral and cellular immunity, the induced immunosuppressive agents targeting T cells are being recognized as currently the most effective medicine (**Table 1**).

**Table 1:** The application of immune inducing therapeutics in islet transplantation.

Generic name	Trade name	Mechanism	Ref.
Antithymocyte globulin (ATG)	Thymoglobulin	Polyclonal antibody, profound T-cell depletion	(32)
Muromonab-CD3	Orthoclone OKT3	Anti-CD3 mAb, T-cell depletion	(33)
Alemtuzumab	CampathLemtrada	Anti-CD52 mAb, T-cell depletion	(34)
Basiliximab	Simulect	Anti-CD25 mAb IL-2 receptor antagonist	(35)
Daclizumab	Zenapax	Anti-CD25 mAb IL-2 receptor antagonist	(5)
Anti-CD154-mAb	–	Blockage of CD40/CD154 T-cell costimulation	(36)

In addition, several new inhibitors have shown promising induction of immune tolerance potential. Alpha-1 antitrypsin (AAT) is a component of serum, synthesized in the liver and secreted into the blood; it is a crucial serine protease inhibitor. Recent studies have shown that AAT can suppress IFN-γ induced M1 macrophage activation/polarization by suppressing STAT1 phosphorylation and inducible nitric oxide synthase (iNOS) release. The results indicated that AAT could inhibit cytokines or dying islets induced macrophage activation, thereby improving islet survival. In addition, in recipients receiving islets and AAT, 20 of 29 reached normoglycemia, compared to only 10 of 28 in those receiving islets only, at 60 days post-transplantation (37).

Ubiquitin-editing protein A20 functions as a negative regulator of immunostimulatory factors (38). It is essential for controlling signals, including the activation of nuclear factor-κB transcription factors, which might be an ideal gene therapy candidate for islet transplantation (25). Chu et al. demonstrate that loss of A20 in B cells can cause an inflammatory syndrome with autoimmune manifestations in old mice (39). Zammit et al. designed an islet cell line that can overexpress A20 through an adenoviral vector encoding human A20 and transplanted the modified cells beneath the kidney capsule in diabetic C57BL/6 mice. The results suggested that the overexpression of A20 will reduce inflammation and prolong the survival of grafts without immunosuppression (38).

The ultimate goal of all immunosuppressive agent intervention is by preventing the allo-/auto-immunity rejection to transplanted islets to preserve their function and maintain stable glucose. Although considerable effort has been devoted to this field, a signal long-term effective therapy has not been identified. Refinement and a combination of the immunosuppressive agents mentioned above may potentially prolong the duration of glycemic control.

### Islet Encapsulation

**Figure 3:**
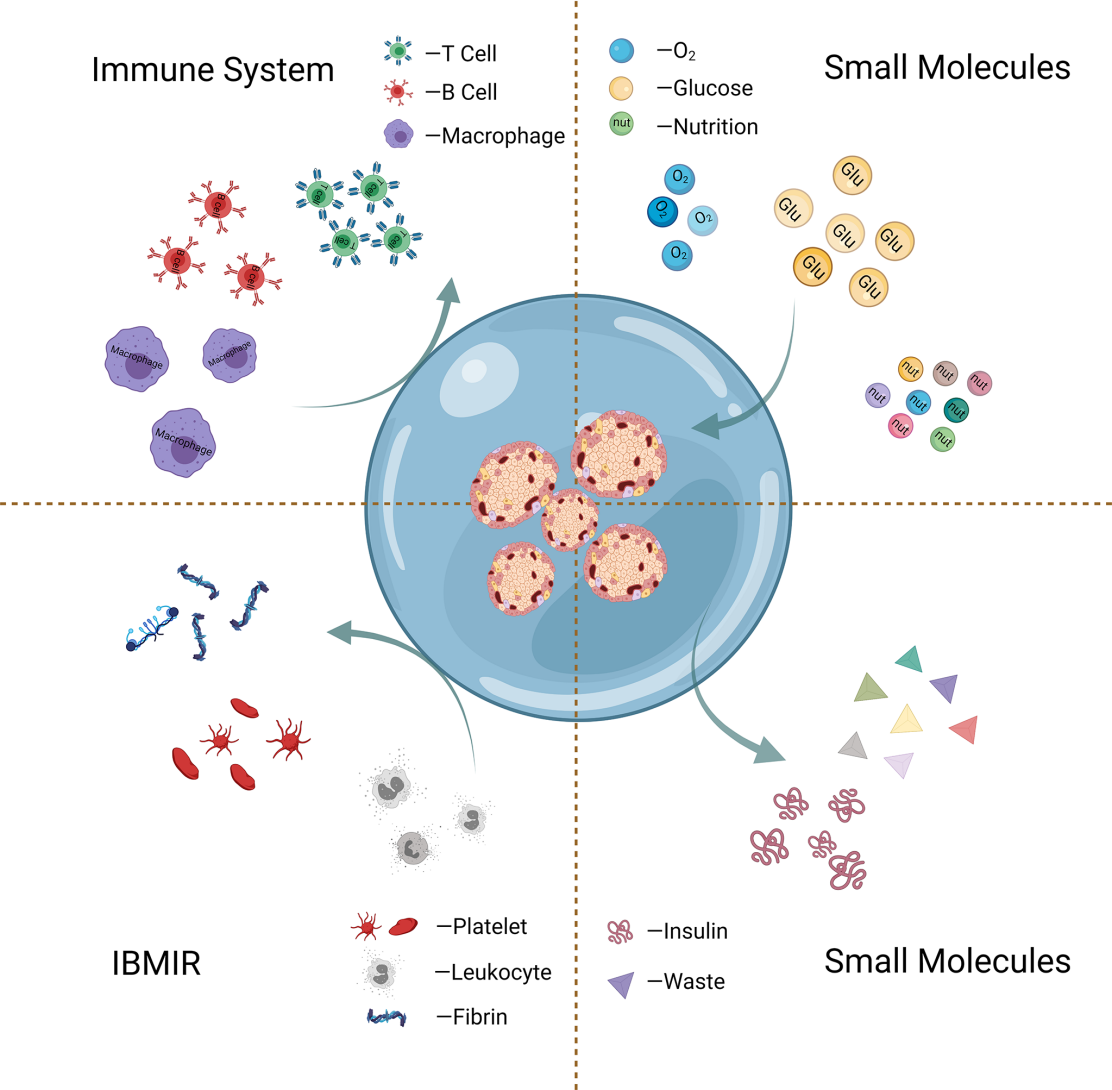
Schematic graph of islet encapsulation that allows substance (oxygen, glucose, etc.) exchange and simultaneously acts as a physical barrier to prevent the transport of immune cells and harmful large molecules.

Hyperacute rejection (HAR), where host antibodies target the antigens presented on the surface of cell graft, has been proposed as the largest contributor to the immediate rejection of cellular graft (40). The principal aim of islet encapsulation is to isolate the cells from the host by a physical barrier, as presented in **Figure 3**. The encapsulating walls act as a selective barrier that prevents the transport of immune cells and large molecules (e.g., antibodies and complements) of the host immune system, which can directly or indirectly injure the grafted cells. It should be pointed out that the developed outer barrier should allow the timely bi-directional diffusion of small molecules such as oxygen, glucose, insulin, and nutrients critical for islets between the islets and the host (41).

One of the first examples of islet encapsulation to treat diabetes was transferring human insulin-secreting tissue with a membranous bag into rats in 1933. Until the early 1950s, a series of experiments studied and compared the survival rate of allotransplanted tissue with or without cell-impermeable encapsulating membrane, and the fields of immune-isolated islet encapsulation technique were established (42). These studies confirmed that the use of the surface encapsulation technique could prevent immune cells from directly contacting and activating the antigen presentation pathway and protect islet grafts from immune attacks.

With the goal of creating immune-protected β cells, various encapsulating approaches have been developed over the past decades. Recent research on islet encapsulation has broadly divided into two directions. One is more traditional, which aims to create physical barriers by biomaterials-based encapsulation to directly reduce the contact between immune cells and islets, thus protecting islets from immune attacks. Another one seeks to optimize the grafted microenvironment of islet graft for improved islet survival and function by *in situ* release of therapeutics or oxygen.

#### Physical Barrier to Prevent Immune Attack

As mentioned above, it is difficult to find a suitable permeable material with precise porosity, good chemical/mechanical stability, and low immunogenicity (43). Therefore, the design and modification to chemical properties, size, and coating mode of encapsulating biomaterial have become the focus of pure physical isolation.

*Microencapsulation.* Microencapsulation approach is about developing immunoprotective microsized capsules coated with biomaterials for encapsulation of cells. In 1964, the cell microencapsulation technique was first described by Chang et al., and until 1980, an alginate–polylysine–polyethyleneimine microcapsule-based microencapsulation technique was first applied into islet transplantation, resulting in prolonged islet survival and normoglycemia in diabetic receivers (42). Over the next several decades, research focused on designing materials with biocompatibility for microencapsulation. As previously mentioned, the biomaterial used for islet transplantation could also induce host response and cause the formation of fibrous capsules, thus impairing the transport functions of the selective permeable encapsulating membrane. Usually, natural polymers are preferred owing to their mild properties, with alginate as the most predominantly used due to its biocompatibility, non-degradability, adjustable stiffness, and also its controllable pore size of the formed membrane to prevent cell infiltration. Alginate can form a hydrogel system *via* ionic crosslinking with a divalent cation such as Ca^2+^ and Ba^2+^. Ca^2+^ is used more often for alginate gelling due to its non-toxicity, while Ba^2+^ could form a more robust hydrogel (44). Remarkable progress has been achieved in alginate microencapsulated islets or stem cell-derived β cells to reverse hyperglycemia. The stem-cell-derived β cell encapsulated by alginate led to the restoration of normoglycemia in immune-competent diabetic mice for 90 days (45). However, conventional alginate might induce foreign body reaction (FBR), which results in fibrotic deposition, nutrient isolation, and donor tissue necrosis. Alginate modification is a popular method of improving various aspects of islet transplantation. Vegas et al. reported that triazole-thiomorpholine-dioxide-modified alginate-encapsulating β cells derived from human pluripotent stem cells have immunosuppressive properties, allowing sustained normoglycemia glucose responsiveness for over 174 days in immune-competent diabetic C57BL/6J mice without immune suppression, even at the end of the experiment. Implants retrieved after the observation period contained viable insulin-producing cells (44).

*Macroencapsulation.* Despite the potential of microencapsulation, one main issue regarding alginate capsules is the difficulty to retrieve or replace them after implantation due to the complicated tissue structure and the large capsule number required for a human patient (46). Macroencapsulation devices (>1mm) may suit this need greatly. The macroencapsulation device incorporates islets into a selectively permeable membrane, which evades the immune response, enabling insulin delivery from the grafts (47). In the 1990s, Baxter Healthcare designed a planar pouch featuring a bilaminar polytetrafluorethylene membrane system named Theracyte™ device. The outer layer promotes tissue integration, where the inner membrane has a 0.4-µm pore size that possesses cell impermeable property. Many studies have used this device in diabetes research to protect transplanted islets from immune rejection. Gabr et al. found that the Theracyte capsule protected the xenogenic IPCs (human stem cells transplanted in diabetic dogs) from host immune rejection and prolonged the cell function duration, even for 18 months. After removal of the Theracyte capsules, fasting blood sugar levels of dogs quickly returned to pretransplantation readings, further confirming the viable islet function (48). In addition, Kirk et al. transplanted the Theracyte™ device encapsulated human embryonic stem cells (hESCs) to mice subcutaneously. Their results suggested that this encapsulation device restricted direct contact between grafts and host cells, allowing further maturation of transplanted hESCs (49–51). ViaCyte Inc. created a macroencapsulation device termed Encaptra™, which has an outer plastic wave support matrix and an inner thin immune barrier layer to protect grafts. In 2017, ViaCyte launched the second trail using perforated macroencapsulation containing PEC-01 cells, in which cell survival will be improved by more optional neovascularization, but recipients in the trail will require full systemic immunosuppression (52). Skrzypek et al. developed a novel multibore system using non-degradable polyethersulfone (PES) blending with polyvinyl-pyrrolidone (PVP). This equipment showed excellent oxygen permeability over a large number of implanted human islets (6,000) within 7 days. The glucose-induced insulin secretion test further confirmed the maintenance of the endocrine function of the implanted cell (53).

In most macroencapsulation devices, the permeability will be considered, but the avoidance of islet clumping is often overlooked, increasing the oxygen diffusion distance and islet death (47). The shape and material of the device need to be modified to solve this problem. Stephens et al. designed subcutaneous injectable collagen oligomers encapsulation for islet loading. The oligomer matrices exhibited improved mechanical stability and resistance to proteolytic degradation compared with monomeric collagens (54). The glucose-stimulated insulin secretion (GSIS) curves and immunostaining results confirmed that the oligomer-based macroencapsulated islets have better cytoarchitecture and phenotype than the free islets. Their subsequent 90-day study also indicated that collagen oligomer-based biomaterials possessed strong immunoprotective properties and successfully prevented islet aggregation after transplantation (55). Interestingly, An et al. also developed a different method for the same anti-aggregation purpose (56). Inspired by the spider, this group designed a highly wettable, Ca^2+^ releasing non-porous polymer thread, which could promote the *in situ* formation of alginate hydrogel around the thread. In their short-term research, the device successfully transported rat islets into immunocompetent C57BL/6 mice and provided sufficient immunoprotection (56). The blood glucose level of diabetic recipients decreased to the normal range within 2 days, and the mice remained euglycemic until the device was retrieved. To prove the scalability and retrievability of this device, this group also performed some large animal experiments using dogs. At 1 month post-transplantation, the device could be quickly and easily retrieved by a minimally invasive laparoscopic procedure, indicating its clinical translational potential.

*Nano-coating Encapsulation*. The current most common site is the intraportal site in clinic (5), with the islets infusing into the hepatic microcirculation. An ideal encapsulation technique for clinic intraportal islet transplantation must therefore be suitable for intraportal delivery to the capillary bed, thus excluding macrocapsules or microcapsules due to their relatively big size. In contrast, nano-coating generates a biocompatible nanometer-sized encapsulating wall, which could ensure that the encapsulated islets remain in a small size and can be implanted into any site (57). Farooq Syed et al. coated isolated human islets with multilayer nano-encapsulation made from chitosan and poly (sodium styrene sulfonate) the reduction of glycemia was faster and quantitatively stronger in nano-encapsulation islets than in uncoated islets after transplantation under the kidney capsule of diabetic mice (58). Alginate is also used in nano-encapsulation due to its significant advantages. Zhi et al. used alternate layers of phosphorylcholine-derived polysaccharides (chitosan or chondroitin-4-sulfate) and alginate as nano-coating materials to encapsulate islets (59). In a syngeneic mouse model, no deleterious response to the coating was observed, and more importantly, the nano-encapsulated islets effectively reversed hyperglycemia. During the 1-month monitoring period, five of the seven mice retained the function of nano-encapsulated islets after allotransplantation. The results showed that the nano-scale encapsulation offers localized immune protection for implanted islets and can limit the early allograft loss.

#### Functionalized Encapsulation Layer to Modulate Immune Microenvironment

Pure physical encapsulation could protect the graft from the direct attacks of the recipient’s immune system, but the direct immune attack is not the only problem for the transplanted islets. Hypoxia stress, inflammatory cytokine attacks, delayed revascularization, and lack of nutrients could all contribute to the death of the graft and cause severe related immune reactions. Thus, the encapsulation layer can be endowed with additional functions such as drug delivery; the local application of immunosuppressive agents can significantly reduce the dosage and directly enhance local immune tolerance. This combination of extra therapeutic drugs and resistant barriers might better protect encapsulated islets and improve overall graft survival (57).

Oxidative stress plays a crucial role in activating alloreactive and autoreactive immunity toward the engrafted islets (60). Therefore, encapsulating islets with biomaterials that possess antioxidant properties might delay or relieve immune-mediated rejections. Barra et al. generated nanothin encapsulation materials (TA-PVP) for islet protection, which is composed of tannic acid (61), a polyphenolic compound with ROS scavenging and anti-inflammatory activities, and poly(N-vinylpyrrolidone). mRNA analysis demonstrated that the TA-PVP encapsulation increases the expression of the anti-inflammatory gene Arg1 and decreases the expression of proinflammatory chemokines Ccl2, Ccl5, and Cxcl10, confirming the ability of TA-PVP to promote the anti-inflammatory innate immune phenotype and elicit localized immunosuppression. The intraperitoneal glucose tolerance test (IPGTT) curves of the TA-PVP encapsulation group were glucose responsive and similar to that of healthy mice. At the same time, islet-only grafts failed to achieve effective glycemic control, indicating that TA-PVP encapsulation successfully protected islets from immune reactions and contributed to glucose homeostasis. Bilirubin is an endogenous metabolic end-product of heme catabolism, which has the ability of anti-inflammation, antioxidative, and immune modulation (62). The protective effect of bilirubin on T1D and islet transplantation has been widely reported (63–65). Zhao et al. developed an ϵ-polylysine-bilirubin conjugate (PLL-BR) to encapsulate the islets (66). The encapsulation matrix increased the production of superoxide dismutase (SOD) and reduced glutathione (GSH) and decreased the expression of MDA and LDH. In addition, the reduced expression of M1 markers (iNOS and CD86) and the increased expression of M2 markers (CD 206 and Arg-1) in the PLL-BR-treated group indicated that the PLL-BR could regulate macrophage polarization effectively. After transplantation of PLL-BR encapsulate islets in diabetic mice, the recipients maintained normoglycemic for 5 weeks, 2 weeks longer than the animals with untreated islets. Therefore, therapeutic-drug-included encapsulation strategies could more efficiently protect the islets from hypoxia-induced oxidative stress, especially in the early stage post-transplantation.

**Figure 4:**
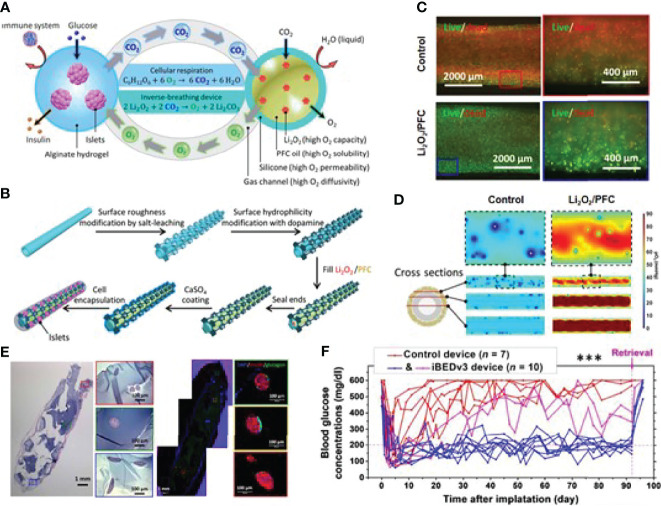
An inverse-breathing system-based islet encapsulation. **(A)** Schematic illustration of the inverse-breathing system: CO_2_ released from semipermeable alginate-hydrogel-encapsulated islets transported to PFC-encapsulated Li_2_O_2_ particulates, converted to O_2_. **(B)** Fabrication of Li_2_O_2_/PFC-containing constructs. **(C)** Fluorescent microscopy images for the viability of INS-1 cells culture in a hypoxic environment with different treatments. **(D)** pO_2_ distributions in three cross-sections of each design. White regions represent necrosis. **(E)** H&E and immunohistochemical staining of retrieved islet graft over 3 months. **(F)** Non-fasting blood glucose measurements after islet transplantation over 92 days. Reproduced, with permission, from (69) Copyright ^©^ 2021 The Authors.

Adequate oxygenation of the transplanted islet remains challenging (67). *In situ* oxygen production and exogenous oxygen supply are two main methods to address the inadequate oxygenation issue. Thus, it is beneficial to combine the *in situ* oxygen generation strategy into the islet encapsulation technique. Coronel et al. developed an oxygen-generating device based on calcium peroxide, which is hydrolytically active to generate oxygen *via* the chemical reaction without enzyme. After transplantation, 100% of the recipients transplanted with the device achieve euglycemia with a mean time of 7 days, compared to the 50% of animals receiving implants only with a delayed reversal time at 14 days post-transplantation (68). *In situ* oxygen production is undoubtedly smart, but recycling waste into oxygen could be an attractive and economical alternative. Wang et al. presented an encapsulation system that could generate oxygen from their own waste product CO_2_ in a self-regulated way (69). The gas–solid (CO_2_–lithium peroxide) reaction that generates O_2_ based on the chemical reaction between Li_2_O_2_ and CO_2_ was utilized to make that happen, as indicated in **Figure 4**. They encapsulated 500 islet equivalents (IEQ) of rat islets and transplanted them into the dorsolateral subcutaneous space of STZ-treated diabetic C57BL6/J mice. As shown in **Figure 4**, normoglycemia was achieved in 8 of 10 inverse-breathing encapsulated-islet-treated mice and maintained for 92 days, whereas all control subjects went back to being hyperglycemic at 30 days after transplantation. On day 92, the retrieved islets maintained smooth and intact morphology, suggesting that the islet was properly preserved. This group also studied the potential of this inverse-breathing oxygen-generation technique in a larger animal model that xenotransplant rat islets into Göttingen minipigs. The islet grafts were retrieved after 1 month and examined to indicate the subrenal microenvironment with or without oxygen generation. As the result shown, most islets presented as fragmented or necrosed in the control group, revealing that the subcutaneous space is challenging for islet survival. In contrast, the inverse-breathing encapsulated islets showed a high survival rate, and most of them presented a healthy morphology after retrieval at 1 month. This result demonstrated that inverse-breathing encapsulation contributed to the generation of oxygen and improvement of anoxic microenvironment.

The islets are highly vascularized within the pancreas, which is crucial to maintain their capacity to secrete insulin quickly in response to elevated blood glucose. The isolation procedure breaks the connections between systemic circulation and the islet vasculature. It results in significant ischemic and mechanical injury, leaving islets more susceptible to post-transplant stress. Pre-vascularization of the encapsulation system has been proven to help isolated islets accommodate the transplanted site by accelerating the vascularization process. For example, Weaver et al. developed a vasculogenic polyethylene glycol (PEG) encapsulation hydrogel for islet delivery to the extrahepatic islet transplant site. This system consists of an S–S cross-linked PEG hydrogel core and a proteolytically degraded vasculogenic outer layer (67). Once transplanted, the encapsulation system could be enzyme-responsively degraded, significantly enhancing localized vascularization and re-implementing the blood supply for transplanted islets. The increased vascular density contributes to higher oxygen tension, which is beneficial for the survival and function maintenance of cells in the device. This hypothesis was proved to be correct by both whole-mount confocal and lectin perfused cross-sectional imaging after 4 weeks. Additionally, finite element analysis confirmed the increased oxygen concentration after pre-vascularization treatment, with an average central oxygen tension within the gels of 0.027 and 0.018 mM for vasculogenic PEG hydrogel and non-vasculogenic PEG hydrogel, respectively.

The inconsistent long-term efficacy hindered the translation of transplanted encapsulated islets to treat T1D in humans. The encapsulation can act as an immune barrier and deliver adjuvant drugs to solve various graft problems post-transplantation. It has been pointed out that one of the major challenges for islet encapsulation is the biocompatibility issue, which could generate a severe inflammatory response. A drug loading encapsulation system could successfully prevent early inflammation post-transplantation. For example, Maurizio et al. developed a ketoprofen-loaded islet encapsulated system to prevent early loss (70). Biodegradable microspheres containing ketoprofen were enveloped into the well-established alginate/poly-L-ornithine/alginate capsules through the layer-by-layer method. Their results indicated that this islet microsphere had high biocompatibility and the ability to reduce inflammatory reactions and pericapsular fibrotic overgrowth altogether.

Islet encapsulation has achieved leapfrog progress because of advances in material sciences, nanotechnology, and pharmaceutical sciences. In particular, the functionalized encapsulation that provides additional properties could endow isolated islets with enhanced survival and function *in vivo*, especially in the early stages of post-transplantation. Those easy-to-make biomaterials with multi-functions were very competitive in the views of clinical translation. In addition, continuous advances in materials and immunology might inspire more alternatives for islet encapsulation techniques.

### Scaffold Aided Islet Transplantation

The peri-islet extracellular matrix (ECM) is a scaffold of fibrillary proteins, accessory proteins, and molecules that provide structural and biological support for surrounding pancreatic islets. The peri-islet ECM provides cell anchorage and signaling that are critical for the islet’s glucose responsiveness. Growing evidence shows that ECM not only acts as homeostatic support for pancreatic islets but also provides physical and immunological barriers against immune infiltration (71). Generally speaking, scaffolds engineered for islet transplant are made of biomaterials that provide mechanical support for the islets and simulate the pancreatic microenvironment. The scaffold improves the islet viability and function by promoting cell adherence and nutrient diffusion and providing ECM-mimicking support (72). In addition, scaffolds can also deliver therapeutic drugs to the implanted site. Thus, it is feasible to include immunosuppressive agents into the scaffold system and modulate early immune reactions that are directly against newly implanted islets.

The selection of biomaterials for scaffold building is crucial for successful islet transplantation. The ideal biomaterials must not cause apparent toxicity, inflammation, or even severe host response while at the same time providing enough mechanical support (73). For example, Smink et al. studied and compared the uses of three different Food and Drug Administration (FDA)-approved polymer candidates, including poly(D,L-lactide-co-e-caprolactone) (PLCL), poly(ethylene oxide terephthalate)/polybutylene terephthalate (PEOT/PBT), and polysulfone in islet transplantation (74). Culture on PEOT/PBT and polysulfone profoundly did harm to islets and induced severe tissue responses *in vivo*. PLCL was the only polymer that could sustain the cell function and survival. In a Rowett nude rat model, after transplantation of 3,500 islets in the PLCL scaffold, the hyperglycemia reversed within 3 days. Poly (lactide-coglycolide) (PLG) is also an FDA-approved biodegradable material that has been developed to build clinical drug/cell delivery (75). Biomeier et al. fabricated PLG scaffolds as a synthetic microenvironment for islets and found out the islet-PLG scaffold transplanted onto intraperitoneal fat maintained euglycemia for over 200 days in the diabetic mice (76). PLG scaffold could be modified or loaded with therapeutic drugs to achieve versatile functions to support islet graft better. Skoumal et al. designed a FasL chimeric with streptavidin-functioned PLG scaffold (77). *In vivo* data indicated that this modified PLG scaffold successfully prolonged islet graft survival from 23 days (transient rapamycin-treated) to over 200 days when the Balb/c islets in the scaffolds were transplanted into the peritoneal fat of diabetic C57BL/6 mice. Liu et al. also used a PLG scaffold for islet transplantation, and their group further explored the loading of IL-33 to achieve localized immunomodulatory in transplanted mice (78). Compared to the untreated group, the median survival time of allogeneic grafts with IL-33 loading scaffold increased from 14 to 33 days. Some other polymers such as poly(glycolic acid) (PGA), poly(lactic acid) (PLA), polycaprolactone (PCL), and other syntheses have also been reported as scaffold materials for islet delivery. These materials are primarily used as solid scaffolds to provide mechanical stability (79).

Besides the mechanical support, it is important to endow more properties to simulate ECM function and provide a favorable microenvironment for islet graft. One direct approach is the use of ECM components, e.g., fibrin and hyaluronic acid. Fibrin is a provisional matrix protein derived from fibrinogen, extensively used as hydrogel material and sealant in clinic (80). Fibrin could bind to surface receptors like integrin and thus act as an effective cell-supportive 3D scaffold by promoting cell differentiation, proliferation, function, and survival. In particular, fibrin has a direct beneficial effect on cultured islets, including islet morphology, insulin secretion, and islet angiogenesis (81). Salama et al. subcutaneously transplanted 5,000 neonatal porcine islets (NPIs) to diabetic immune-compromised mice and studied the beneficial role of fibrin in islet transplantation (82). The grafts transplanted with fibrin achieved euglycemia between 5 and 22 weeks. In contrast, NPIs transplanted alone failed to reverse hyperglycemia under the same condition. At 22 weeks post-transplantation, mice underwent a survival nephrectomy of the graft-bearing kidneys, and then, the animals were all back to being hyperglycemic within 48 h. Fibrin could not only promote the islet survival through its biological activities but also build ECM mimicking cell/drug delivery carrier. Maillard et al. created a fibrin scaffold for islet culture by simultaneously loading perfluorodecalin, an oxygen diffusion enhancing medium (83). Perfluorodecalin was added to increase oxygen diffusion toward isolated islets and improve their function and viability as well. In this study, the scientists assessed cell apoptosis through caspase-3 activation and found out that the apoptosis of the treated group was significantly lower than that of the untreated group, indicating that fibrin matrix supplemented with perfluorodecalin loading can provide a beneficial physical and chemical environment for improved islet *in vitro*. However, fibrin clots will enable the host immune system cells to prevent infection, and many studies have indicated that fibrin can promote macrophage recruitment and cytokine production (80). This immune-mediated response might lead to chronic rejection and is not good for long-term fibrin application in islet grafts. Another concern for fibrin might be its biodegradable feature, making it unknown for the duration of fibrin to support islet *in vivo*.

**Figure 5:**
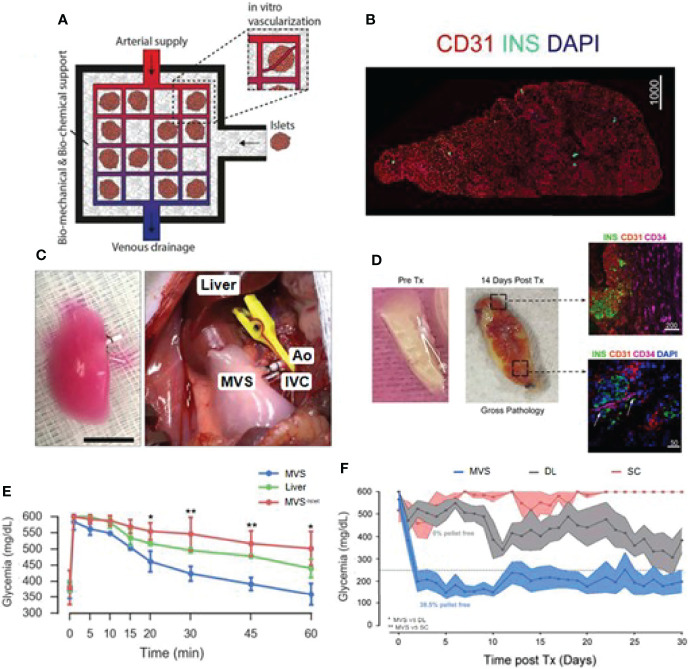
Bioengineered mature vascularized scaffold (MVS) for islet transplantation. **(A)** Schematic illustration of the bioengineered vascularized islet organ for transplantation. (**B)** Image of a MVS’ cross-section after 7 days. **(C)** Left: Images of a mature MVS for transplantation. Right: MVS transplanted into the abdominal cavity. Pulmonary artery and pulmonary veins were connected to inferior vena cava (IVC) and aorta (Ao). **(D)** MVS preTx and 14 days after transplantation. Right top image, CD34^+^ vascular ingrowth very close to islet and human CD31^+^ region. Right down image, murine vascular network was preliminarily established inside MVS at 14 days post-transplantation. **(E)** Blood glucose levels after intravenous glucose tolerance test in diabetic rats 1 h after vascular anastomosis. **(F)** Non-fasting blood glucose measurements after islet transplantation over 30 days. Reproduced, with permission, from (85) Copyright (2019), Elsevier Ltd.

The acellular scaffold is another emerging alternative for islet transplantation. Acellular scaffold usually keeps intact ECM comprising a mixture of structural and functional molecules by removing the allogeneic or xenogeneic cellular antigens from the original tissue during decellularization. The acellular scaffold can be the lair for cell protection and adhesion during the transplantation process. It is an isolated extracellular matrix from the tissues or organs of various species. It can also serve as a promoter of structural and functional repair (84). Citro et al. employed an acellular lung tissue to bio-fabricate functional islet organ, as shown in **Figure 5** (85). To provide a biocompatible multicompartment scaffold, they first pre-vascularized acellular lung matrix prior to islet seeding and obtained mature vascularized scaffold (MVS) and found out that islets could be more easily integrated into the surrounding vasculature after 7 days of culture as compared to the non-vascularized group. In response to elevated glucose levels, MVS could quickly release insulin and provide a more efficient reduction of hyperglycemia than its control counterparts. After transplantation, the MVS group can achieve euglycemia and maintain it for 30 days (**Figure 5**). A similar pre-vascularized acellular scaffold was also reported by Han et al. Their study fabricated this scaffold by coating islet-seeded fibrin hydrogen onto decellularized human umbilical arteries (86). This mini-equipment allows oxygen-rich arterial blood to flow through and provides a more islet-friendly microenvironment. When implanted, it enables restoration of normoglycemia for 90 days in the receiving diabetic nude rats. Some scientists believed that whole organ decellularization might provide a better option and generate a more pancreas-similar condition for grafted islets. Large animal studies should be conducted while exploring the use of the acellular organ for clinical application.

Scaffolds are designed to resemble the natural organs and imitate their function. An insulin-secreting bio-organ might be a perfect option. However, the choice of composite-biocompatible and scaffold architecture design still largely hurdle the further development of bio-organ scaffold in islet grafts, especially considering its long-term survival and function.

### Choosing an Immune-Privileged Grafted Site

**Figure 6:**
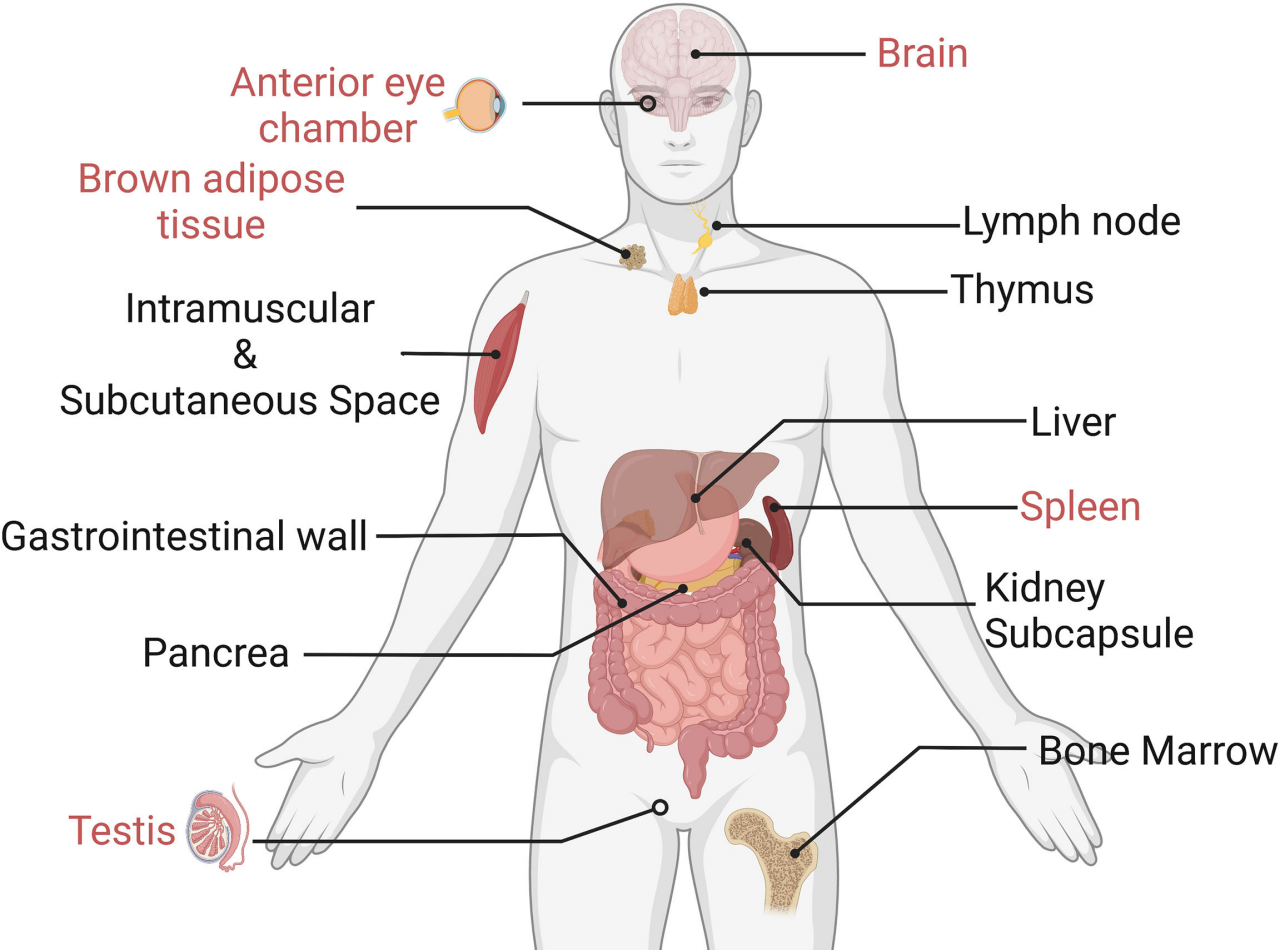
Experimental islet transplant sites (the immune-privileged sites are marked in red). Only immune-privileged sites will be reviewed in this paper.

An optimal grafting site with a long-term grafting feature should be given for islet transplantation. Ideally, this site should offer venous drainage portals to enable blood glucose levels to be stabilized. In addition, the potential site should provide grafts with similar oxygen tension as the pancreas did. Some scientists pointed out that the site should also supply easy access to post-transplant islet functional and morphological monitoring (87). In terms of the immune response, grafted sites should have limited exposure to blood and immune cells to prevent inflammatory reactions. Actually, an ideal location that meets all the requirements is yet to be identified (88). We have listed some recent emerging grafted sites for islets in **Figure 6**. In the following part, we would like to introduce their own merits and demerits, with an emphasis on those that could avert immune reactions.

The liver (portal vein) is currently the preferred site for islet transplantation, constituting about 90% of clinical islet grafts. One of the major reasons to select the liver in clinical islet grafts is its procedure feasibility. Liver islet transplantation could be done through a minimally invasive approach without the need for surgery. Additionally, portal veins also enable efficient insulin delivery to avoid systemic hyperinsulinemia (88). However, the intrahepatic islet transplantation’s long-term survival and efficacy are limited due to the liver-specific complications, such as islet infusion into the bloodstream will trigger IBMIR, which can damage intraportal transplanted islet. Additional intraportal islet transplantation complications might include portal thrombosis and hypertension. Portal thrombosis is a life-threatening complication. Portal hypertension might raise the post-transplant bleeding risk, portal thrombosis, and the occurrence of sepsis (89).

Recently, an immunologically privileged site was constantly mentioned for islet transplants. The brain, the testis, and the anterior eye chamber are organs that inhibit the immune response and are thus known as privileged immune locations. In such sites, immune responses are largely or completely suppressed, avoiding many immunological problems that islet grafts once faced. These immune-privileged sites revisualized islet transplantation and offered a valuable occasion for expanding the survival of the allograft. However, their immune privilege mechanisms are not clearly understood. The immunological peculiarities of these sites may result from a combination of causes. For instance, the blood barrier in the retina, brain, and testis are kept immunosuppressed because of the physical cellular shield (90), while the regulatory T cells (Tregs) provide immune privilege under some circumstances as well.

The anterior chamber of the eye (ACE) has been proposed as an optimal islet implantation site. The eyes have always been considered as an immune-privileged site, which might relate to the immunosuppressive state in the anterior chamber associated with immune deviation and the tolerance related to regulatory T cells. Not only that, the ACE also provides implanted islets with an oxygen-rich milieu, directly alleviating the hypoxia of newly implanted islets. In an experimental study, allogeneic islets were transplanted into the anterior chamber of the right eye of a diabetic recipient baboon, followed by an anti-CD154 antibody (an immunosuppressor) therapy (90). Results showed that the intraocular islet allografts were retained for >400 days without subsequent immunosuppression. Furthermore, the ACE could be a novel imaging site in diabetes research for observing transplanted cells activity, since the eyes are inherently an optical apparatus (91); it allows undisturbed imaging with unparalleled penetration depth and resolution. Therefore, the status of islet graft in ACE could be monitored in real time so that appropriate immune intervention could be carried out promptly. So far, islet transplantation to the anterior chamber of the eye has been approved by FDA for clinical trial (92). However, considering the discomfort and potential effects on vision, recipients are limited to those diabetic patients with at least one eye with extensive vision loss from hand motion to no light perception. Meanwhile, some researchers are worried that transplanted islets in the ACE site are still vulnerable to autoimmunity, reminding us that immunomodulation might still be required (93).

The testis has been suggested as an immune-privileged location for islets (88). It has been reported that intratesticular islet transplantation resulted in good metabolic function that is capable of maintaining euglycemia in rats (94) and shows delayed rejection in both allograft and xenograft. Nasr et al. used a testicular islet allotransplanted model and revealed that the islet transplanted into the testis generates fewer CD8+ memory cells but induces more specific CD4+CD25+ Treg cells than that in a more conventional site (renal capsule) (95). In addition, blocking CD40/CD40L costimulation could cause intratesticular islets’ immune tolerance, which is not observed in the renal subcapsular islets. These findings demonstrated that the testis is superior to inducing transplantation tolerance as an immunologically privileged site over the traditional location, e.g., renal subcapsule. While the testis may not accept a large number of islets to restore normoglycemia (96), testis islet transplantation could be conducted as a pioneering procedure to induce peripheral tolerance and protect a second site that can receive enough size of the graft.

For insulin delivery into the brain, transplanting islets into the cranial subarachnoid cavity was often exploited. The immune-privileged property and excellent nutrient supply make the subarachnoid cavity of the brain a feasible transplantation site. Most intracranial islet transplantation is operated to ameliorate cognitive impairment and peripheral metabolic dysfunctions. Bloch et al. first developed a rat model with severe dementia associated with obesity and cerebral amyloid-β angiopathy and then transplanted 100 islets into the cranial subarachnoid space (97). During the 6-month post-grafting period, the grafted islets significantly improved cognitive functions in recipients. A similar study was also conducted by Konstantin et al. (98). They transplanted the cells into the subarachnoid cavity surrounding the olfactory bulb to reverse diabetes and cognitive dysfunction. All diabetic rats achieved normoglycemia within the 2 days after receiving 3,000 IEQs and maintained it for over 2 months. Additionally, the histological results confirmed that grafted islets still preserved complete architecture after 2 months. Considering the risk of craniotomy and the difficulty of the clinical application, the brain, as an alternative transplantation site, is still under debate and needs more in-depth studies.

As an organ that is responsible for immune tolerance, the spleen is considered as immunosuppressed. The splenic T cell was reported to include suppressor T cells, which prevent dendritic cells from presenting antigens to effector T cells and suppress the proliferation of effector T cells *via* the expression of suppressive cytokines IL-35 and IL-10. Choosing the spleen as a transplant site could also reduce the islet quantity required to achieve euglycemia. Ltoh et al. studied and compared the islet numbers that are needed to achieve normal blood glucose in diabetic mice receiving islets at three different transplant sites, namely, the liver, kidney, and spleen (99). The *in vivo* data indicated that all diabetic mice gradually became normoglycemic after transplanting 50 islets into the spleen surface. The marginal number for the spleen (50) was half that for the kidney (100) and less than half that for the liver (200). The advantages of the spleen might be attributed to the physiological insulin drainage and regulation of immunity. Additionally, some researchers believed that the spleen might also be one source of islets, with splenic cells potentially differentiating into insulin-producing cells (100). Splenic mesenchymal stem cells have also been reported to repair the damaged tissues and promote the regeneration of pancreatic islets (101).

Brown adipose tissue (BAT) could maintain thermogenesis by converting the energy into heat (102). BAT is fully vascularized, which could provide transplanted islets with sufficient oxygen and nutrient. More importantly, BAT contains rich activated M2 macrophages and Tregs, therefore displaying an overall anti-inflammatory condition. These immune-regulated cells are beneficial for islet engraftment by dampening inflammatory immune response after transplantation. Xu et al. demonstrated that islets transplanted into BAT of STZ-induced mice could restore euglycemia and maintain health glucose metabolism for over 1 year (103). After removal of islet-engrafted adipose tissue, the average blood glucose levels of diabetic mice went up to over 500 mg/dl immediately within 1 day. Kepple et al. further explored the effects of BAT islet transplantation on BAT function and immune system in recipient mice (104). Quantitative real-time PCR (qRT-PCR) data indicated no change in BAT-specific mRNA encoding Adrb3, Zic1, and the critical, thermogenic, uncoupled protein Ucp1, suggesting that the islet transplantation does not affect energy expenditure and thermogenesis of BAT. Meanwhile, islet transplants into BAT significantly delayed immune-mediated graft rejection in an allograft model. However, how exactly the human adipose tissue will affect the application of BAT as a transplantation site is still unknown. Further studies that characterize BAT mass and BAT transplantation operability should be conducted to explore BAT as a more clinically relevant graft region.

### Cell Therapy

Cell therapy has emerged as a promising alternative to replace or enhance the biological function of damaged tissues using autologous or allogeneic cells. Generally speaking, islet transplantation could also be classified as one kind of cell therapy (105). Here, we focused on cell therapy that involves other cells that assist islets with their own biological functions to better survive in the transplanted site. In the following part, we will introduce some immunomodulatory cells, which have been investigated to suppress or delay immune reactions to improve the successful operative rate of islet transplantation.

#### Mesenchymal Stem/Stromal Cells

Mesenchymal stem/stromal cells (MSCs) are non-hematopoietic multipotent stromal cells. The ability of MSCs to secrete trophic and angiogenic factors can help early grafts rebuild vascularization after transplantation (106). Moreover, MSCs can utilize extracellular matrix as structural support and also as a bioactive molecular container. The various functions of MSC make it an attractive candidate to protect cells in islet transplantation. MSCs help the woe of grafts by targeting the major causes of post-transplantation failure—hypoxia and immune rejection. Recent studies indicated that MSCs transfer mitochondria to islets during *in vitro* co-culture, which rescue the cells from hypoxia (107). In addition, MSCs could secrete a large number of bioactive molecules that potentially affect immune and inflammatory reactions. For example, MSCs might block the differentiation of monocytes into DCs and also impair their antigen-presenting ability (108). This phenomenon may explain the profound immunosuppressive effects of MSCs on virtually any component of the immune system. Therefore, MSCs have been utilized in islet transplant to improve the overall islet survival, especially for allograft. For example, Kenyon et al. reported that MSCs and allogeneic cynomolgus monkey islets co-transplantation into the liver portal vein of the diabetic cynomolgus monkey recipient successfully prolonged allogenic islets from 24 to 81 days by increasing Tregs numbers in the periphery (109). Ishida et al. found that the MSC co-transplanted with islets intraportal transplantation inhibited the NK cells in the liver by secreting prostaglandin E2 (110) and markedly improved the islet survival.

Although MSC-assisted islet transplantation has been widely reported in a variety of animal models, most of the research mainly focused on the effect of MSCs on alloimmunity. It is still unclear whether MSCs could impede recurrent autoimmunity. In-depth knowledge of the immunomodulatory mechanisms of MSCs might help to address these concerns and promote the future application of MSCs incorporated islet transplantation in clinic.

#### Dendritic Cells

As part of the immune response, DCs can be critical to achieving central and peripheral tolerance. Thus, DCs could be a potential therapeutic target in the design of tolerogenic regimes (83). While mature myeloid DCs unregulated MHC class II and CD40, CD80, and CD86 costimulatory molecules, immature DCs could downregulate these markers that are active allospecific T-cell response inhibitors (111). The absence of stimulatory molecules enables immature DCs to cause particular hypo-responsiveness of antigen in T cells. Tolerogenic DCs have been shown to help allograft adoption through deletion of alloreactive T cells and activation of donor-specific regulatory T cells (Treg), and skewing the Th1/Th2 response (112). Although many studies exploit DCs as an immune target, few studies reveal its potential in islet co-transplantation. Long et al. grafted rat islets together with mouse mesenchymal stem cells (MSCs) and/or immature DCs into diabetic mice (113). The data suggested that the transplantation with either MSCs or immature DCs is better to control blood glucose levels compared to the transplantation of islets alone. In addition, co-transplantation of islets together with MSCs and immature DC obtained better results and significantly enhanced islet grafts to reverse hyperglycemia in mice with T1D. However, as for DC-assisted islet transplantation, the generation and maintenance of tolerogenic DCs remain a problem.

#### Regulatory T Cells

Tregs is a small and unique subset of CD4+ T cells that comprises approximately 1%–10% of normal adult peripheral blood. Tregs play a vital role in maintaining immune homeostasis and regulating inflammatory disease progressions. They suppress the inappropriate immune responses to self-antigens, such as those occurring in T1D. Emerging evidence suggests that Tregs dysfunction might be a cause of T1D (114). Thus, those immunomodulators and cell therapies that target Tregs are considered to be of prodigious long-term potential. Yi et al. studied the effect of Tregs therapy on islet xenotransplantation (115). They transplanted neonatal porcine islets into the NOD-SCID IL2rγ−/− mice treated with or without Treg injection afterward. Treg injection treatment delayed the rejection of xenografts from 28 days (without Tregs injection) to 100 days (with Tregs injection) through a potent suppression of a predominantly CD4+ T-cell-mediated pathway. Immunohistochemical analysis indicated that no visible insulin-positive staining cells presented in the non-treated xenografts, while intact insulin-positive staining cells could be clearly observed in treated grafts (115). Co-transplantation of Tregs and islets was also studied by Naohiro et al. In their study, Tregs from C57BL/6 mice and islets from Balb/c mice were made into aggregates and loaded on agarose hydrogel with small round-bottomed wells before intraportal transplanted into C57BL/6 diabetic mice. No systemic immunosuppression was used post-transplantation. Their results suggested that Tregs in the aggregates enable six of nine transplanted grafts survival for more than 100 days (116), substantially increasing long-term allografts survival. The use of Tregs in islet transplantation is still in its infancy and needs further exploration. Tregs-related tolerance signatures need to be refined and optimized for individualized patients.

Additional cell-assisted islet transplantation could be promising. However, these cell-based strategies described above were only tested in animal models. Concerns regarding immunological compatibility, how to master multi-types of cell delivery, and general quality control and safety issues need to be addressed before moving cell therapy forward in islet transplantation application.

## Conclusion

Islet transplantation has proven its long-term efficacy during the past decades. However, donor shortage and post-transplantation immune response limit its widespread use. With the development of stem cell and genetic modification technologies, it becomes possible to provide an unlimited number of insulin-producing cells. Thus, how to protect grafted islets without the long-term use of systemic immunosuppression has become a focused research area (117). In this paper, we introduced the immune responses against the transplanted cells and summarized recent progress in formulations and process strategies to provide immune protection for improved survival and function of transplanted islets.

A joint strategy that combines biomaterial-based encapsulation/scaffold with local immunomodulation has proven its potential in islet transplantation. This combo could protect islet graft from host immune rejection and IBMIR, and act as carriers of immunosuppression agents or assistant cells. Synthetic biomaterials are relatively easy to prepare and meet quality control requirements during mass production as safe “non-living” products. However, they have their own issues, including biocompatibility, durability, and the potential to trigger foreign body responses, which still need to be fully addressed before clinical application.

From the immunological perspective, the ideal graft sites should have restricted immune responses and ensure the lowest rate of islet loss. The so-called immune-privileged sites (e.g., brain, testis, anterior eye chamber) could meet this requirement (118). Nonetheless, these promising transplanted sites are relatively hard for surgical operation and post-transplantation monitoring. In addition, there is little experience in large animal models and human trials, making the side effects and long-term efficacy of islet transplant at the immune-privileged site rather uncertain.

Co-transplantation with immunomodulatory cells could also be a promising approach. Once transplanted, functional immune-regulating cells could act as drug reservoirs and produce cytokines and growth factors to assist co-transplanted insulin-producing cells on demand. However, problems such as maintenance of their immunoregulatory function and longevity of the cells are needed to be considered. Additionally, these cells are rare cell types and not easy to collect. In addition, further efforts should be made to ensure the stability, potency, and retention of these assisting immunomodulatory cells after the islet graft procedure.

In summary, we reviewed various immune-protective formulation and process strategies for improved survival and function of transplanted islets. The single method mentioned above cannot alleviate the dilemma faced by islet transplantation, while combining them might create long-term functional and safe cell therapies for T1D. The ultimate goal of islet transplantation is to completely cure diabetes without needing long-term immunosuppressive therapy. Although the task is challenging, success is possible.

## Author Contributions

Conception and design of study: QY, LK. Drafting the manuscript: YS, ZW, QW, LK, and QY. Revising the manuscript critically for important intellectual content: Y-ZZ, ZJ. All authors contributed to the article and approved the submitted version.

## Funding

This research was supported by the National Natural Science Foundation of China (Grant No. 81903551), Zhejiang Medical and Health Science and Technology Program (Grant No. 2022492641), Zhejiang provincial program for the cultivation of high-level innovative health talents (Y-ZZ), 151 talent project of Zhejiang province and 551 talent projects of Wenzhou (Y-ZZ) and Excellent Young Scientist Training Program fund from Wenzhou Medical University.

## Conflict of Interest

The authors declare that the research was conducted in the absence of any commercial or financial relationships that could be construed as a potential conflict of interest.

## Publisher’s Note

All claims expressed in this article are solely those of the authors and do not necessarily represent those of their affiliated organizations, or those of the publisher, the editors and the reviewers. Any product that may be evaluated in this article, or claim that may be made by its manufacturer, is not guaranteed or endorsed by the publisher.
